# Clostridium difficile Infection: Risk and Poor Prognostic Factors at a Tertiary Hospital in the Eastern Region of Saudi Arabia

**DOI:** 10.7759/cureus.39193

**Published:** 2023-05-18

**Authors:** Mohammed A Miqdad, Kranthi Kosaraju, Abdullah Mohamad, Hasan Hulwi, Ubaid Rais, Mohammad Taleb, Talal Aloreibi

**Affiliations:** 1 Internal Medicine, Dr. Sulaiman Al Habib Medical Group, Khobar, SAU; 2 Tele-Geriatric Research Fellowship, Michigan State University, Michigan, USA; 3 Microbiology, Dr. Sulaiman Al Habib Medical Group, Khobar, SAU; 4 Pharmacology, Dr. Sulaiman Al Habib Medical Group, Khobar, SAU; 5 Infectious Diseases, Dr. Sulaiman Al Habib Medical Group, Khobar, SAU

**Keywords:** infectious diarrhea, proton pump inhibitor, nosocomial infection, pseudomembranous colitis, clostridium difficile infection

## Abstract

Background: *Clostridium difficile* (*C. difficile*) is a common cause of hospital-acquired diarrhea. It is associated with significantly higher mortality and morbidity in addition to the cost-effectiveness burden on the healthcare system. The primary risk factors for *C. difficile* infection (CDI) are past *C. difficile* exposure, proton pump inhibitors, and antibiotic usage. These risk factors are also associated with poor prognosis.

Objective: This study was performed in Dr. Sulaiman Al Habib Tertiary Hospital in the Eastern Region of Saudi Arabia. The aim was to evaluate the risk and prognostic factors of CDI and their association with the outcomes of hospital stay, such as complications, length of stay (LOS), and treatment duration.

Patients and methods: This is a retrospective cohort study for all patients who tested for *C. difficile* in the medical department. The target population was all adult patients ≥16 years with positive stool toxins for *C. difficile* between April 2019 and July 2022. The main outcome measures are risk and poor prognostic factors for CDI.

Results: *C. difficle* infection patients were included in the study; 12 (52.2%) were female, and 11 (47.8%) were male. The mean age of the patients was 58.3 (SD: 21.5) years; 13 (56.5%) patients were below 65 years, and 10 were above 65 years. Only four patients were without comorbidities, and 19 (82.6%) patients had various comorbidities. Importantly, hypertension was the most common comorbidity in 47.8% of the patients. Furthermore, advanced age significantly impacted the hospital LOS as the mean age among patients who stayed at the hospital less than four days and those who stayed ≥4 days was 49.08 (19.7) and 68.36 (19.5), respectively (*P* = .028).

Conclusion: Advanced age was the most frequent poor prognostic factor among our inpatient participants with positive CDI. It was significantly associated with longer hospital LOS, more complications, and longer treatment duration.

## Introduction

*Clostridium difficile* (*C. difficile*) is an anaerobic, gram-positive, spore-forming bacillus and the most frequent cause of infectious diarrhea [1]. Due to antimicrobial disruption of the colon's natural microbiota, this bacterium may cause gastrointestinal tract infections. The majority of nosocomial diarrheal infections in hospitalized patients have been correlated to *C. difficile* infection (CDI), posing serious risks to global public health due to increased morbidity and death. Several toxins are produced and released in connection with CDI symptoms [2-4].

Watery diarrhea, nausea, fever, and stomach discomfort are all signs of CDI. Nevertheless, clinical signs of CDI can range from asymptomatic carriers to moderate diarrhea to severe fulminant infection with sepsis, toxic megacolon, and transmural pancolitis that may need colectomy [5]. Although patients with inflammatory bowel disease and those confined to critical care units have much greater mortality rates, the overall mortality rate for CDI is 2% [6] In 2011, there were about 29,000 fatal CDI infections and close to 500,000 cases overall in the United States [7]. There are limited data on CDI in Saudi Arabia; however, a similar study to ours in the Western Region of Saudi Arabia had shown a prevalence rate of 6.8% [8].

Adults over 65 represent the great majority of CDI fatalities; in 2008, CDI was the 18th highest cause of death in this age group [6-7]. The fecal-oral pathway is the route of *C. difficile* transmission. Healthcare institutions are most at risk of spreading infections because of contaminated air, surfaces, and staff members. Therefore, surface cleaning and proper personal hygiene among healthcare personnel are crucial to decrease the risk of CDI, and CDI patients must also be isolated to prevent transmission. The CDI probability is increased by several variables, including hospitalization, antibiotic exposure, and advancing age [1,9]. The United States recorded an 8% decrease in CDI between 2011 and 2014; numerous nations have implemented protocols and guidelines to reduce CDI in the acute-care setting through antibiotic stewardship, outbreak management, case detection and appropriate contact precautions, personal protective equipment, and environmental cleaning [5,10,11].

The two primary risk factors for CDI are past *C. difficile* exposure and antibiotic usage. In addition, immunocompromised persons, irritable bowel syndrome, chronic kidney illness, extended exposure to healthcare institutions, and enteral feeding (tube feeding) can increase the risk of CDI. In addition, after a few studies looked at the relationship between *Helicobacter pylori* treatment and CDI, using a proton pump inhibitor (PPI) in the two months before diagnosis increased the risk of CDI. Numerous further investigations were conducted after this relationship between PPI usage and CDI was discovered, although the results and interpretations have been contradictory [1,5,11]. In our study, we aim to present the characteristics of our patients with CDI and evaluate the factors associated with poor prognosis and outcomes.

## Materials and methods

This study design was a retrospective cohort conducted in Dr. Sulaiman Al Habib Hospital, a tertiary medical center in Al Khobar City, Saudi Arabia. The test used to diagnose *C. difficle* was a rapid immunochromatography assay for detecting *C. difficle* toxin in stool specimens using a commercial kit supplied by Meridian Biosciences. The target population (inclusion criteria) was all adult patients ≥16 years with positive CDI between April 2019 and July 2022. Out of 1,123 tested patients, 23 came back positive for CDI. Exclusion criteria were any patient who did not meet any of the aforementioned requirements.

All the patients' characteristics, comorbidities, and potential risk factors were obtained retrospectively from the electronic medical records. Variables included patient demographics (age, gender), past medical history of hypertension, diabetes mellitus, and other comorbidities, such as cardiac and inflammatory bowel diseases, neurological/mental disorders, and previous CDI. Other variables include medication history and prognostic factors such as complications, length of stay (LOS), and duration of treatment, as in Table 1. Data entry was managed using Microsoft Excel (Microsoft, Washington, United States). Then, it was analyzed using SPSS Statistics (IBM Corp. Released 2021. IBM SPSS Statistics for Windows, Version 28.0. Armonk, NY: IBM Corp.). Categorical variables were presented as frequencies and percentages and compared using the chi-square test. However, Fisher’s exact test was used when there were less than five counts in any [mM1] of the cells. An independent t-test was used when there were continuous variables. The data was presented with a 95% confidence interval, and a p-value of < 0.05 was statistically significant.

**Table 1:** Demographic and medical data of the included patients (n=23) CDI: *C. difficile* infection, PPI: proton pump inhibitor, LOS: length of stay

Data	Number	
Age [mean (SD)]	58.3 (21.5)	
Age groups	≤64	13 (56.5%)
	≥65	10 (43.5%)
Presence of comorbidities	No	4 (17.4%)
	Yes	19 (82.6%)
Diabetes mellitus	No diabetes mellitus	14 (60.9%)
	Diabetes mellitus	9 (39.1%)
Hypertension	No hypertension	12 (52.2%)
	Hypertension	11 (47.8%)
History of cardiac disease	No	19 (82.6%)
	Yes	4 (17.4%)
Presence of mental disorder	No	17 (73.9%)
	Yes	6 (26.1%)
Presence of inflammatory bowel disease	No	19 (82.6%)
	Yes	4 (17.4%)
History of CDI	No	20 (87%)
	Yes	3 (13%)
History of antibiotic use	No history of antibiotics	9 (39.1%)
	Antibiotics used before	14 (60.9%)
History of PPI use	No	8 (34.8%)
	PPI	15 (65.2%)
Antibiotics treatment of CDI	No CDI treatment	1 (4.3%)
	Metronidazole	4 (17.4%)
	Vancomycin	14 (60.9%)
	Vancomycin and metronidazole	4 (17.4%)
CDI treatment with vancomycin	No vancomycin	5 (21.7%)
	Vancomycin	18 (78.3%)
Hospital LOS	≤3	12 (52.2%)
	≥4	11 (47.8%)
Duration of CDI treatment	7-10 days	14 (60.9%)
	≥14 days	8 (34.8%)
Complications	No	20 (87%)
	Yes	3 (13%)
Mortality	No	23 (100%)
	Yes	0

## Results

Twenty-three patients with CDI were included in the study, where 12 (52.2%) were female and 11 (47.8%) were male. As presented in Table 1, the mean age of the patients was 58.3 (SD: 21.5) years; 13 (56.5%) patients were below 65 years old, and 10 were above 65 years of age. Only four patients were without comorbidities, and 19 (82.6%) patients had different comorbidities, such as diabetes and hypertension. Importantly, hypertension was the most common comorbidity being present in 47.8%. Diabetes is the second most common comorbidity being present in 39.1%. Cardiac diseases (such as coronary artery disease and heart failure), mental disorders (such as dementia and history of cerebrovascular events), and inflammatory bowel diseases were also present in 17.4%, 26.1%, and 17.4%, respectively. Only three (13%) patients reported a previous CDI. Fourteen (60.9%) patients reported prior antibiotics use, and nine (39.1%) reported not. Regarding PPIs use, 65.2% have a history or prior PPIs use, and 34.8% have not.

Regarding the hospital course, 12 (52.2%) patients stayed for three days or less, and the rest (47.8%) had four or more LOS. The majority of the patients (60.9%) received vancomycin, and only four patients received metronidazole. Four patients also received a combination of metronidazole and vancomycin for the treatment of CDI; hence, the use of vancomycin was reported in 18 (78.3%) patients. The duration of antibiotics treatment was 7-10 days in 14 (60.9%) patients and ≥14 days in eight (34.8%) (one patient did not use any antibiotics). Complications were developed in only three patients; one developed acute kidney injury, another experienced metabolic acidosis, and the other developed only mild (grade I) hypokalemia (3.4 mmol/L). The mortality rate was 0 in our study.

Furthermore, advanced age significantly impacted the hospital LOS as the mean age among patients who stayed at the hospital less than four days and those who stayed ≥4 days was 49.08 (19.7) and 68.36 (19.5), respectively (P = .028). In addition, longer hospital LOS (≥4 days) was shown to be associated with a history of previous cerebrovascular accidents or dementia (P = .0045). In addition, prior PPIs use was associated with longer LOS (P = .0271). Regarding antibiotics management, vancomycin was used more among patients with a longer hospital LOS ≥4 days (P = .0372) (Table 2).

**Table 2:** The relationship between the patients’ characteristics and hospital LOS CDI: *C. difficile* infection, PPI: proton pump inhibitor, LOS: length of stay

Data		Hospital LOS		p-value		
		≤3 days	≥4 days			
Age [mean (SD)]		49.08 (19.704)	68.36 (19.587)		0.028583	
Age groups	≤64	9 (39.1%)	4 (17.4%)		0.099533	
	≥65	3 (13%)	7 (30.4%)			
Presence of comorbidities	no	3 (13%)	1 (4.3%)		0.590062	
	yes	9 (39.1%)	10 (43.5%)			
Diabetes mellitus	No diabetes mellitus	7 (30.4%)	7 (30.4%)		1	
	Diabetes mellitus	5 (21.7%)	4 (17.4%)			
Hypertension	No hypertension	7 (30.4%)	5 (21.7%)		0.536809	
	Hypertension	5 (21.7%)	6 (26.1%)			
History of cardiac disease	No	10 (43.5%)	9 (39.1%)		1	
	Yes	2 (8.7%)	2 (8.7%)			
Presence of neurological/mental disorder	No	12 (52.2%)	5 (21.7%)		0.004577	
	Yes	0	6 (26.1%)			
Presence of inflammatory bowel disease	No	9 (39.1%)	10 (43.5%)		0.590062	
	Yes	3 (13%)	1 (4.3%)			
History of CDI	No	11 (47.8%)	9 (39.1%)		0.590062	
	Yes	1 (4.3%)	2 (8.7%)			
History of antibiotic use	No history of antibiotics	6 (26.1%)	3 (13%)		0.400323	
	Antibiotics used before	6 (26.1%)	8 (34.8%)			
History of PPI use	No	7 (30.4%)	1 (4.3%)		0.027191	
	PPI	5 (21.7%)	10 (43.5%)			
CDI treatment	No CDI treatment	1 (4.3%)	0		1	
	Metronidazole	4 (17.4%)	0			
	Vancomycin	6 (26.1%)	8 (34.8%)			
	Vancomycin and metronidazole	1 (4.3%)	3 (13%)			
CDI treatment with vancomycin	No vancomycin	5 (21.7%)	0		0.037267	
	Vancomycin	7 (30.4%)	11 (47.8%)			

Regarding complications, the mean age of the patients who developed complications was 80.33 (7.63) years; meanwhile, the mean age of those who did not develop any complications was 55 (21.08) years (P = .0556). Importantly, all three patients who developed complications received a combination of vancomycin and metronidazole to treat CDI (P = .0009) (Table 3).

**Table 3:** The relationship between patients’ characteristics and complications CDI: C. difficile infection, PPI: proton pump inhibitor

		Complications		p-value
		no	yes	
Age [mean (SD)]		55 (21.089)	80.33 (7.638)	0.055673
Age groups	≤64	13 (56.5%)	0	0.067758
	≥65	7 (30.4%)	3 (13%)	
Presence of comorbidities	no	4 (17.4%)	0	1
	yes	16 (69.6%)	3 (13%)	
Diabetes mellitus	No diabetes mellitus	13 (56.5%)	1 (4.3%)	0.537549
	Diabetes mellitus	7 (30.4%)	2 (8.7%)	
Hypertension	No hypertension	11 (47.8%)	1 (4.3%)	0.590062
	Hypertension	9 (39.1%)	2 (8.7%)	
History of cardiac disease	No	17 (73.9%)	2 (8.7%)	0.452851
	Yes	3 (13%)	1 (4.3%)	
Presence of mental disorder	No	16 (69.6%)	1 (4.3%)	0.15528
	Yes	4 (17.4%)	2 (8.7%)	
Presence of inflammatory bowel disease	No	17 (73.9%)	2 (8.7%)	0.452851
	Yes	3 (13%)	1 (4.3%)	
History of CDI	No	18 (78.3%)	2 (8.7%)	0.356296
	Yes	2 (8.7%)	1 (4.3%)	
History of antibiotic use	No history of antibiotics	8 (34.8%)	1 (4.3%)	1
	Antibiotics used before	12 (52.2%)	2 (8.7%)	
History of PPI use	No	8 (34.8%)	0	0.525692
	PPI	12 (52.2%)	3 (13%)	
CDI treatment	No CDI treatment	1 (4.3%)	0	0.000944
	Metronidazole	4 (17.4%)	0	
	Vancomycin	14 (60.9%)	0	
	Vancomycin and metronidazole	1 (4.3%)	3 (13%)	
CDI treatment with vancomycin	No vancomycin	5 (21.7%)	0	1
	Vancomycin	15 (65.2%)	3 (13%)	

Moreover, the mean age of the patients treated for 7-10 days and ≥14 days until infection resolution was 52.93 (21.38) and 72.13 (12.64), respectively (P = .032). Interestingly, hypertension was significantly associated with a longer duration of treatment (P = .0237) (Table 4).

**Table 4:** The relationship between patients’ characteristics and duration of treatment CDI: C. difficile infection, PPI: proton pump inhibitor

Data		Duration of CDI treatment		p-value
		7-10 days	≥14 days	
Age [mean (SD)]		52.93 (21.381)	72.13 (12.643)	0.032012
Age groups	≤64	10 (45.5%)	2 (9.1%)	0.074303
	≥65	4 (18.2%)	6 (27.3%)	
Presence of comorbidities	no	4 (18.2%)	0	0.253589
	yes	10 (45.5%)	8 (36.4%)	
Diabetes mellitus	No diabetes mellitus	10 (45.5%)	3 (13.6%)	0.186997
	Diabetes mellitus	4 (18.2%)	5 (22.7%)	
Hypertension	No hypertension	10 (45.5%)	1 (4.5%)	0.023736
	Hypertension	4 (18.2%)	7 (31.8%)	
History of cardiac disease	No	12 (54.5%)	6 (27.3%)	0.601914
	Yes	2 (9.1%)	2 (9.1%)	
Presence of mental disorder	No	12 (54.5%)	4 (18.2%)	0.136504
	Yes	2 (9.1%)	4 (18.2%)	
Presence of inflammatory bowel disease	No	11 (50%)	8 (36.4%)	0.272727
	Yes	3 (13.6%)	0	
History of CDI	No	13 (59.1%)	6 (27.3%)	0.527273
	Yes	1 (4.5%)	2 (9.1%)	
History of antibiotic use	No history of antibiotics	5 (22.7%)	3 (13.6%)	1
	Antibiotics used before	9 (40.9%)	5 (22.7%)	
History of PPI use	No	5 (22.7%)	2 (9.1%)	1
	PPI	9 (40.9%)	6 (27.3%)	
CDI treatment	Metronidazole	3 (13.6%)	1 (4.5%)	0.704149
	Vancomycin	8 (36.4%)	6 (27.3%)	
	Vancomycin and metronidazole	3 (13.6%)	1 (4.5%)	
CDI treatment with vancomycin	No vancomycin	3 (13.6%)	1 (4.5%)	1
	Vancomycin	11 (50%)	7 (31.8%)	

## Discussion

Aging and CDI

Advanced age was the most frequent poor prognostic factor among our participants. It was responsible for longer hospital LOS, more complications, and longer duration of treatment. In the published literature, age is also a significant risk factor for the development of CDI. Older people are more likely to acquire CDI and tend to have severer outcomes [12-14]. In Negrut et al., the average age of CDI patients was roughly 69 years old, and there was no statistically significant difference between the two groups being monitored [15]. In a similar study on 877 CDI patients, the age group 55 to 74 was most impacted by CDI [16].

According to published research, people over 65 years of age had a five- to tenfold higher chance of developing CDI compared to patients under 65 [17-19]. However, a sizable fraction of CDI affects a younger population, and a substantial risk factor for CDI itself and for poor clinical outcomes, including severity and death, is age > 65 [17-19]. Similar to our study, patients below 65 years of age have better clinical outcomes in general despite no significant statistical difference. In comparison to the control group, there were considerably more case group members over 65. Not only is this age group referred to as a risk factor for CDI, but patients in this age range also have a poor prognosis, which increases clinical severity and mortality rate [17-19].

Several processes might explain the latter behavior; First, a poor innate or humoral immune response may increase the likelihood and severity of CDI; second, the greater frequency of CDI in the elderly may be linked to changes in the intestinal microbial composition, such as the loss of bacterial diversity that comes with aging and may facilitate *C. difficile* colonization [20-22]. In addition, this age group is considerably more prone to chronic diseases, infections, and subsequent polypharmacy, particularly antibiotics [23]. The majority of CDI patients were between the ages of 60 and 69, which is the usual age at which individuals begin to undergo immunological senescence and changes in the composition of their gut microbiota [24].

Besides, the Th2-type immune response, which is more efficient against external pathogens and toxins and is driven by interleukins 4 and 5, is suppressed by decreased estrogen production. The development of CDI is also significantly influenced by the human microbiome, which changes secondary to aging. In the human microbiota, 99% of the bacteria were *Firmicutes* and *Bacteroidetes*, and it is well-known that their ratio declines with aging [25-26].

Dementia and CDI

Similar to our findings, Negrut et al.'s paper showed that dementia triples the chance of a CDI recurrence (OR = 3.26, 95% CI = 1.26-8.41, P = .014) [15]. While it has not yet been determined if dementia and CDI recurrence are correlated, the prevalence of dementia with aging may rise due to environmental variables, stress, vitamin deficiencies, endocrine problems, structural brain damage, physical causes, and other related illnesses. Accordingly, people with dementia are commonly abandoned in care facilities or are bedridden, making them more susceptible to acquiring health deficiencies/infections, followed by antibiotics use or other dysbiosis-related drugs. Further, it is generally known that the vagus nerve, neurotransmitters, neurohormones, and the immune system mediate the link between the gut and the brain, also known as the gut-brain axis. Therefore, chronic brain inflammation, which underlies depression, is also a leading cause of altered gut permeability and microbiota composition via cytokines. It is worth mentioning that the gut flora may also be altered due to the medications used for mental illness [27-28].

PPI and CDI

Prior use of PPIs is a significant risk factor for the development of CDI [29]. In our study, almost two-thirds of patients (65.2%) have a previous history of PPI use. In addition, PPI use was associated with longer hospital LOS as two-thirds of patients who have used PPIs previously stayed four days or more, and one patient of non-PPI users stayed more than four days (P = .0271). The role of PPIs in the emergence of CDI is still up for debate, given that some studies failed to show an association between stomach acid suppression and an increased risk for the emergence of CDI [30]. While the normal amount of gastric acid acts as a defense mechanism for the host, a rise in gastric pH may lead to difficulty evacuating the swallowed *C. difficile* spores [30].

Further prospective studies are required to explore the potential association between prior PPI use and the development of asymptomatic *C. difficile* colonization or CDI, as PPI use is expanding internationally. Numerous investigations were conducted after this association between PPI use and CDI was discovered, although the results and interpretations have been contradictory, as we mentioned earlier [31]. There have been few prospective trials, although one small, perhaps underpowered, multicenter randomized controlled study comparing pantoprazole to placebo in 91 intensive care unit patients failed to detect a statistically significant difference in CDI incidence [32]. The significant heterogeneity constrains the validity of these analyses, such as variable definitions of PPI use (definitions range from three days to any use within the last year), lack of identification of a specific PPI and/or dose, duration of PPI exposure, and numerous potential confounding factors at the patient level [33].

PPIs cause several physiological changes that might raise the risk of CDI, such as bacterial overgrowth, increased gastrin synthesis, increased somatostatin release, and reduced hydrochloric acid production in the stomach. Multiple adverse effects, such as hypomagnesemia, vitamin B12 deficiency, small-intestinal bacterial overgrowth, osteoporosis-related fractures, acute and chronic kidney disease, pneumonia, and diarrheal illness, are correlated to the multifactorial disruption of the gastrointestinal environment and drug metabolism as a result [34-35].

Antibiotics use and CDI

In our research, 60.9% of our patients have a history of antibiotic use, such as Ceftazidime, Ceftriaxone, Cefixime, Augmentin, Clindamycin, Vancomycin, Ciprofloxacin, Meropenem, and Piperacillin-avibactam. Although using any antibiotic increases the risk of CDI, this also includes vancomycin and metronidazole, which are used to treat CDI. Contrary to other antibiotics, broad-spectrum penicillin, cephalosporins, clindamycin, and fluoroquinolones are known to increase the risk of CDI development [18-19]. This positive correlation's rationale is that CDI begins when the usual gut flora comes into close contact with antibiotics. As a result, the normal intestinal microbiota becomes disrupted, which creates an ideal environment for *C. difficile* to grow and spread [36]. The appropriate use of antibiotics is a crucial component of CDI prophylaxis. Thus, when considering antibiotic use, CDI's benefit and risk ratio must be weighed against one another. More importantly, isolating suspected or infected individuals in the inpatient setting are crucial [1,12].

Limitations

The small sample size, lack of a control sample, and single-center design of this retrospective study may lead to recall bias.

## Conclusions

Advanced age was the most frequent poor prognostic factor among our inpatient participants with positive CD. It was significantly associated with longer hospital LOS, more complications, and a longer duration of treatment. We recommended implementing strict hand hygiene, particularly hand washing before and after encounters with those populations during their inpatient stay. This might aid in preventing *C. difficile* transmission through health professionals and subsequent undesirable consequences. Additionally, we suggest conducting further multi-central studies to establish the incidence and prevalence of CDI in Saudi Arabia.
